# Molecular Mechanism of Alzheimer’s Disease

**DOI:** 10.3390/ijms242316837

**Published:** 2023-11-28

**Authors:** Gerhard Rammes

**Affiliations:** Department of Anaesthesiology and Intensive Care Medicine, Medical School, Klinikum Rechts der Isar, Technical University Munich, 81675 Munich, Germany; g.rammes@tum.de

Neurodegenerative disorders are a major public health concern. Despite decades of research, we are still seeking an efficient cure for these pathologies. Alzheimer’s disease (AD), which is closely associated with aging, is the most common neurodegenerative disease and the leading cause of dementia worldwide.

AD, characterized by profound deficits in memory, executive processing, and premature morbidity, represents a major challenge to both the medical community and the broader society. Currently, AD is incurable, and there is no effective treatment. Most cases of AD are sporadic, and very few are inherited. The complex pathological mechanisms of sporadic AD are not yet well understood and remain to be elucidated.

Unfortunately, research in the AD field has faced multiple failures in clinical trials, despite investing tens of billions of dollars. This is, in part, due to the lack of a fundamental understanding of the causes of AD. Another major challenge is the need for further advances in biomarker development, focusing on pathophysiological processes that are important for detection at the early stages of AD disease progression when interventions are likely to be most effective. 

The precise aetiology of AD is known to be complex and multifactorial, with a notable overlap between familial and non-familial forms as well as with different forms of dementia, such as vascular dementia. In AD, the neurodegeneration process involves early synaptotoxicity and loss of neuropil, neurotransmitter disturbances, the accumulation of extracellular β-amyloid (Aβ) deposits (amyloid/senile plaques) as well as intracellular neurofibrils (neurofibrillary tangles, NFTs), gliosis and, only in later stages, the overt loss of neurons and associated brain atrophy [1,2,3]. Additionally, in addition to the two main pathological hallmarks of AD—Aβ peptide and tau protein—current research regarding the pathophysiology and treatment options of AD has been fundamentally extended. In particular, aberrant-activated microglia and neurotoxic astrocytes have been identified, including the involvement of the complement system in synaptic pruning mechanisms and mitochondrial dysfunction that appear to be important mediators, all of which may contribute to synaptic loss and cognitive deficits [4,5]. 

Furthermore, identifying molecular links between Alzheimer’s disease, diabetes mellitus, and other neurodegenerative diseases, which are likely to act synergistically in promoting AD pathology, may help to understand the early and late molecular mechanisms of AD. 

The enormous complexity of the disease, including the prolonged timespan over which AD develops and the initial pathological signs that can be diagnosed, along with its putative multifactorial causality not primarily related to neurodegeneration, justifies the significant efforts currently invested in identifying the mechanisms and fighting AD. 

Drugs, such as anesthetics frequently used in clinical applications, may potentially interact with AD, thereby exaggerating or even triggering its pathophysiology. 

This Special Issue titled “Advance on the Research of Alzheimer’s Disease” of the International Journal of Molecular Sciences includes a total of six original articles and one review article providing new information about research approaches, pathophysiology, treatment options, and drug interactions regarding AD. 

The valuable contributions published in the present Special Issue of IJMS shed more light on many unresolved questions in this field. 

Mingke Song extensively reviewed asparaginyl endopeptidase (AEP) and presents the latest advances in AEP-related neurodegeneration studies and their potential as a modification target for AD [6]. An overactivation of this enzyme may trigger the onset of AD by promoting tau hyperphosphorylation and amyloidosis, leading to synaptotoxicity, neurodegeneration, and cognitive decline. Since these events represent early pathological changes in AD, upregulated AEPs are an emerging drug target for disease modification and a potential biomarker for predicting preclinical AD. 

Two papers in this Special Issue delve into the amyloidogenic pathway and the two crucial enzymes involved, β-secretase BACE1 and presenilin1 (PS1), one of the four subunits comprising γ-secretase. Mutations in the PS1 protein are heavily involved in familiar AD and, interestingly, can also affect BACE1 expression and activity. However, the exact mechanisms are still unclear. Lin et al. (2023) substantiated their hypothesis by using a PS1 knock-out cell line, revealing that PS1 upregulates the distribution and trafficking of BACE1 in the endoplasmic reticulum, Golgi, and endosomes [7]. 

Since β- and γ-secretase initiate the amyloidogenic pathway, significant industrial effort has been directed towards identifying and developing the inhibitors of these enzymes [8,9,10,11]. However, creating secretase inhibitors is challenging because these enzymes have various substrates, such as Seizure protein 6 (Sez6), which is exclusively processed by BACE1 and crucial for dendritic spine plasticity. Furthermore, many secretase inhibitors do not discriminate between BACE1 and its homolog BACE2, thereby producing additional side effects via BACE2. A new treatment strategy would involve using inhibitors that selectively target BACE1. Using longitudinal in vivo two-photon microscopy, Pratsch et al. describe the effects on dendritic spine dynamics induced by different compounds that act as highly selective BACE1 inhibitors [12]. 

The formation of β-amyloid large aggregates is a hallmark of AD and is hypothesized to be the main factor leading to neurodegeneration and hence cognitive decline. In addition to the synaptotoxic effects of Aβ oligomers, membrane-bound oligomers forming Ca^2+^-permeable amyloid pores have been considered to induce excitotoxicity and hence neuronal loss. Therefore, targeting amyloid pores may offer an additional treatment option. The peptide AmyP53 prevents amyloid pore formation by targeting gangliosides, which serve as the plasma membrane receptors of amyloid proteins. Di Scala et al. introduced this peptide and presented promising data regarding its therapeutic potential, which may be administered as a nasal spray formulation to prevent and/or treat Alzheimer’s and Parkinson’s diseases [13]. 

For detailed research on the pathophysiology of AD, the importance of in vitro and in vivo disease models is obvious. Rochin-Fernandez et al. provide a proteome analysis of olfactory ecto-mesenchymal stem cells (MSCs) derived from PSEN1 (A431E) mutation carriers. MSCs possess unique properties that make them a reliable study model for the presymptomatic and symptomatic stages of AD [14]. 

Although obesity and type two diabetes are risk factors for developing AD, recent clinical studies demonstrate that a high body mass index in specific populations is associated with a preventive role in AD [15]. Unfortunately, the relationships and mechanisms between BMI and the risk of AD progression have not been fully elucidated. Using the 5xFAD mouse model, Choi et al. induced obesity in mice by treating them with a high-fat diet. They analyzed the physiological changes in metabolic function and adipogenesis, as well as the pathological changes in the brain, and set these parameters in relation to the pathophysiology of AD. 

The interaction between anesthetics and the pathophysiology of AD is still a matter of debate. However, clinical and preclinical studies are inconsistent and are hindered by factors such as an unambiguous study design or the usage of non-clinical anesthesia regimens. Shi et al. conducted an in vitro study by focusing on astrocyte-dependent synaptic pruning in the presence of Aβ 1-42 and the application of either isoflurane or xenon. In contrast to isoflurane, xenon does not affect GABAergic signaling and also exhibits neuroprotective properties. The presented data demonstrate that both isoflurane and xenon can reverse the Aβ1-42-induced enhancement of synaptic elimination in ex vivo hippocampal brain slices [16].
